# What’s in a Name?

**Published:** 1921-07

**Authors:** 


### WHAT'S IN A NAME?

To those who have witnessed the changes that have taken place in dental practice and the dental attitude of mind for a generation or two, and especially in the case of those who are inclined to subject these passing phenomena to critical psychologic analysis, the results frequently appeal to the sense of humor, assuming that the analyst has a sense of humor, for underlying it all is that combination of attributes collectively designated as human nature, and human nature is always consistently humorous, whatever else it may be. The changes here to be considered are not changes of fact, but rather changes in our mental attitudes toward the group of facts and phenomena with which we are professionally concerned. For example, the earliest records relating to the healing art clearly indicate that tartar and deposits upon the teeth were recognized as contributing causes for disease of the gums and the loosening and ultimate loss of the teeth. Antoni van Leeuwenhoek in 1683 discovered the presence of bacteria in these deposits and stated from his observations that there were "more animals in the human mouth than there were people in Holland."

It was a logical deduction that teeth should be kept free from these harmful deposits, hence the prevalent use from earliest times of the toothbrush, chew sticks and various tooth-cleansing devices, and hence also the practice of dentists who from the beginning of dental history were concerned with the removal of tartar deposits with especially devised instruments and the incidental use of dentifrices for preventing tartar formation.

To sum up this phase of the matter, dentists have cleaned teeth since the foundation of dentistry as an art and have also advised the lay public to keep their teeth clean throughout the same period and for precisely the same elemental reasons that are now generally urged in the prevalent, well-nigh universal, oral hygiene propaganda. But mark the change, not of fact but of mental attitude, toward the fact. Someone adapted the word "prophylaxis" to the tooth-cleaning operation, and presto! the whole operation took on a new significance. There was something mystical about the word "prophylaxis," only a small minority looked up its meaning in the dictionary, many regarded it as the Greek for rubbing the teeth with an orangewood stick armed with pumice powder. At any rate the word had possibilities; it carried a certain dignity and the promise of a better reward for the service rendered. It sounded less like a disagreeable job and more like an operation, more suggestive of a fee or honorarium than of just plain ordinary pay. So we discarded the "menial, scavenger" service of "cleaning teeth'' and embarked on the dignified professional specialty of prophylaxis.

Again, in cases of so-called incurable dento-alveolar abscesses with necrotic apical areas it was early recognized as good dental surgery to cut off the end of the root, bur out the necrotic area until sound tissue was reached, fill the root, pack the cavity of operation with antiseptic gauze and let the cavity heal by granulation. The operation was done as part of the day's work and no particular fuss about it. But again, someone launched it from the docks of operative dentistry into the waters of special surgery by giving it the dignity of a Greco-Latin baptism and rechristening it ''apicoectomy,'' thus speeding it upon its ambitious surgical career with all the implications of skilled specialist, private hospital facilities, trained nurse, and cost to the patient of a laparotomy case.

Moreover, the Hebrew scriptures contain references to dental conditions which strongly indicate that the peoples of that period knew something from experience about pyorrhea alveolaris. At any rate, it is an old story. Poor old pyorrhea! how it has been battered about, maltreated, investigated, distorted, and relabeled out of all resemblance to itself, and that even in the house of its friends. It has had a new name proposed for it by every earnest investigator of its etiology and psychology, but it goes merrily on its course, at the same time giving aid and comfort, not to speak of pecuniary reward, to specialists, non-specialists, nostrum venders, pseudo-philanthropists and, incidentally, no end of grief and trouble to the editors and publishers of dental magazines. Nevertheless, ''there is hope'' from the fact that this disorder or group of disorders once collectively designated almost universally as pyorrhea has also been rechristened with a classic cognomen as ''periodontoclasia,'' and it also will go forth in professional practice to demand a fee for its treatment commensurate in ponderosity with its new appellation. A host of others of the same general character are following in such rapid order that the terminology of dentistry seems doomed to ultimate reconstruction in the near future.

Much may be said in favor of systematic technical terminology in dentistry as in every activity having pretensions to a scientific basis; we will even applaud prophylaxis, apicoectomy, periodontoclasia and the others, if, by reason of these verbal ponderosities, the results relating to the things they designate will thrive and improve *pari passu* with their impressiveness as related to the professional dignity of the respective specialists on the one hand and their fee-compelling influence on the other. It has been argued that this type of terminology is consistent with medical ideals and its use therefore enhances the professional flavor and atmosphere of dentistry; possibly so, yet can it be denied that there is about it a suggestion of pedantry, a straining after effect, therefore a suspicion of disingenuousness that is not wholly compatible with the spirit of scientific truth? Is it not just this lack of directness and simplicity about much of our professional terminology or its implication of mysticism and insincerity that raises a smile in the thoughtful when some of our professional terms are given a public airing?

We want to get this inquiry on record before "cleaning teeth," "root amputation" and "pyorrhea" are classed in our dictionaries as obsolete terms, if only to show that the dental profession successfully did these things before "prophylaxis," "apicoectomy" and "periodontoclasia" were born.—Editorial in *Dental Cosmos*, June, 1921.

